# Phylogenetic Analysis Supports Horizontal Transmission as a Driving Force of the Spread of Avian Bornaviruses

**DOI:** 10.1371/journal.pone.0160936

**Published:** 2016-08-18

**Authors:** Dennis Rubbenstroth, Volker Schmidt, Monika Rinder, Marko Legler, Sönke Twietmeyer, Phillip Schwemmer, Victor M. Corman

**Affiliations:** 1 Institute for Virology, Medical Center–University of Freiburg, Faculty of Medicine, University of Freiburg, Herrmann-Herder Str. 11, D-79104, Freiburg, Germany; 2 Clinic for Birds and Reptiles, University of Leipzig, An den Tierkliniken 17, D-04103, Leipzig, Germany; 3 Clinic for Birds, Reptiles, Amphibians and Ornamental Fish, Centre for Clinical Veterinary Medicine, University Ludwig Maximilian Munich, Sonnenstr. 18, D-85764, Oberschleißheim, Germany; 4 Clinic for Pets, Reptiles and pet and feral Birds, University of Veterinary Medicine Hannover, Bünteweg 9, D-30559, Hannover, Germany; 5 Department of Research and Documentation, Eifel National Park, Urftseestraße 34, D-53937, Schleiden-Gemünd, Germany; 6 Research and Technology Centre Büsum, University of Kiel, Hafentörn 1, D-25761, Büsum, Germany; 7 Institute for Virology, University of Bonn, Sigmund-Freud-Str. 25, D-53127, Bonn, Germany; 8 German Centre for Infection Research (DZIF), Partner Site Bonn-Cologne, Bonn, Germany; Justus-Liebeig University Giessen, GERMANY

## Abstract

**Background:**

Avian bornaviruses are a genetically diverse group of viruses initially discovered in 2008. They are known to infect several avian orders. Bornaviruses of parrots and related species (Psittaciformes) are causative agents of proventricular dilatation disease, a chronic and often fatal neurologic disease widely distributed in captive psittacine populations. Although knowledge has considerably increased in the past years, many aspects of the biology of avian bornaviruses are still undiscovered. In particular, the precise way of transmission remains unknown.

**Aims and Methods:**

In order to collect further information on the epidemiology of bornavirus infections in birds we collected samples from captive and free-ranging aquatic birds (n = 738) and Passeriformes (n = 145) in Germany and tested them for the presence of bornaviruses by PCR assays covering a broad range of known bornaviruses. We detected aquatic bird bornavirus 1 (ABBV-1) in three out of 73 sampled free-ranging mute swans (*Cygnus olor*) and one out of 282 free-ranging Eurasian oystercatchers (*Haematopus ostralegus*). Canary bornavirus 1 (CnBV-1), CnBV-2 and CnBV-3 were detected in four, six and one out of 48 captive common canaries (*Serinus canaria* forma domestica), respectively. In addition, samples originating from 49 bornavirus-positive captive Psittaciformes were used for determination of parrot bornavirus 2 (PaBV-2) and PaBV-4 sequences. Bornavirus sequences compiled during this study were used for phylogenetic analysis together with all related sequences available in GenBank.

**Results of the Study:**

Within ABBV-1, PaBV-2 and PaBV-4, identical or genetically closely related bornavirus sequences were found in parallel in various different avian species, suggesting that inter-species transmission is frequent relative to the overall transmission of these viruses. Our results argue for an important role of horizontal transmission, but do not exclude the additional possibility of vertical transmission. Furthermore we defined clearly separated sequence clusters within several avian bornaviruses, providing a basis for an improved interpretation of transmission events within and between wild bird populations and captive bird collections.

## Introduction

**Fig 1:**
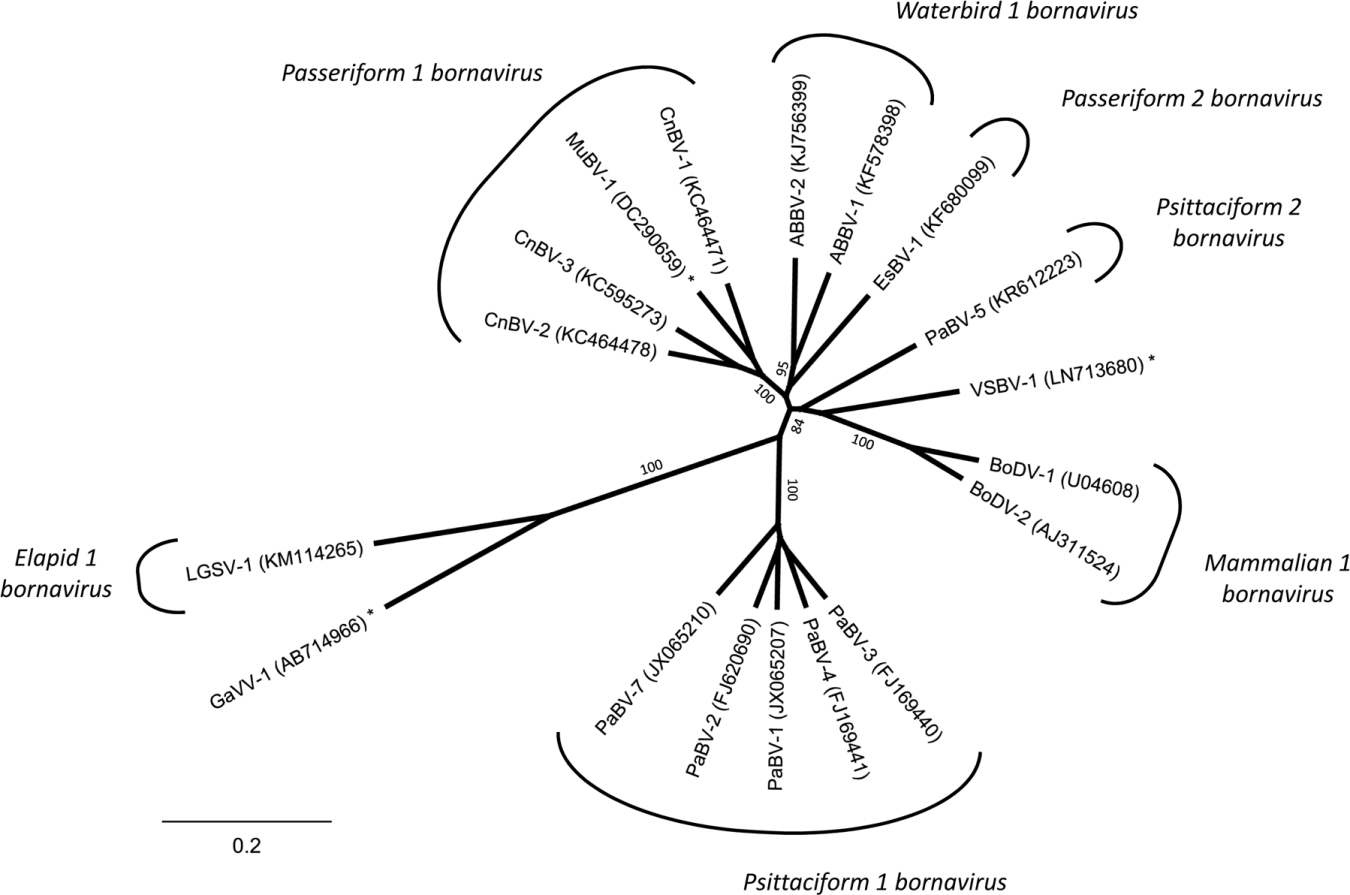
Taxonomy of the *Bornaviridae* family. Complete P gene sequences of representative bornavirus sequences analysed using Neighbor-Joining algorithm and Jukes-Cantor distance model in Geneious R8. Values at branches represent support in 1,000 bootstrap replicates. Only bootstrap values ≥70 at major branches are shown. Bornaviruses marked with asterisks are not classified following the currently accepted taxonomy [2]. ABBV = aquatic bird bornavirus, BoDV = Borna disease virus, CnBV = canary bornavirus, EsBV = estrildid finch bornavirus, GaVV = Gabon viper virus, LGSV = Loveridge´s garter snake virus, MuBV = munia bornavirus, PaBV = parrot bornavirus, VSBV = variegated squirrel bornavirus.

Bornaviruses (genus Bornavirus, family *Bornaviridae*) are enveloped viruses with a negative sense single-stranded RNA genome. To date the family *Bornaviridae* is comprised of at least eight viral species and currently 20 viruses of avian, mammalian and reptilian origin (Fig 1) [1–3]. Bornaviruses replicate in the nucleus and establish persistent, non-cytolytic infections. They preferentially infect neurons, but in host species assumed to serve as natural reservoirs a broad range of other cell types and tissues can be infected and viral shedding occurs via various excretions [4–9].

The majority of the currently known bornaviruses is of avian origin. To date, fifteen genetically highly diverse avian bornaviruses covering six viral species have been described (Fig 1) [1, 2]. Bornaviruses have been detected in a broad range of avian orders, but infections of clinical relevance are found mainly in parrots and their relatives (order Psittaciformes). Parrot bornaviruses 1 to 8 (PaBV-1 to 8) are widely distributed in captive psittacines worldwide [7, 10–14] and epidemiological and experimental results confirmed them to act as causative agents of proventricular dilatation disease (PDD) [5, 6, 11, 12, 15, 16]. PDD is a chronic and often fatal disease characterized by lymphoplasmacytic infiltrations in peripheral and central neuronal tissues. Depending on the location of these lesions, disease manifestations may cover a broad range of clinical signs, including impaired gastro-intestinal function and symptoms of the central nervous system [5, 6, 17].

Furthermore, bornaviruses were detected in several additional avian orders. Captive Passeriformes are natural hosts of canary bornaviruses 1 to 3 (CnBV-1 to 3), munia bornavirus 1 (MuBV-1) and estrildid finch bornavirus 1 (EsBV-1) [8, 18, 19]. Aquatic bird bornavirus 1 and 2 (ABBV-1 and 2) were detected in wild populations of various waterbird species mainly belonging to the order Anseriformes [20–24], while ABBV-1-related viruses were reported from gulls (order Charadriiformes) in North America [25]. In contrast to bornaviruses of parrots, the pathogenic potential of these non-psittacine viruses remains largely unknown.

The knowledge on avian bornaviruses has dramatically increased during the past few years since their first discovery in 2008 [11, 12]. Nevertheless, many important aspects of their biology are still poorly understood. ABBV-1 and ABBV-2 were detected in wild aquatic birds, at least some of which presumably represent the natural reservoir of these viruses [20–24]. In contrast, all other avian bornaviruses known to date were almost exclusively detected in captive birds [8, 10, 13, 14, 18]. Their wild reservoirs still remain unknown and so do their ways of introduction into the captive populations.

Another unresolved issue is the mechanisms of avian bornavirus transmission. Reports of rapidly spreading bornavirus infection and PDD outbreaks in parrot collections suggest horizontal transmission [26, 27]. Due to the detection of infectious bornavirus from cloacal swabs of infected birds, the urofecal-oral route is believed to be a possible transmission route [7, 8, 28]. However, evidence for this hypothesis is poor. Application of bornaviruses to psittacines and common canaries by parenteral injections reliably resulted in persistent infection [5, 6, 8, 15, 16, 29], whereas inoculation via mucosal surfaces did not provide consistent results [8]. Furthermore, under natural and experimental conditions horizontal transmission to contact animals housed in the same aviary appears to be inefficient, further questioning this route of infection [5, 6, 8, 30]. Vertical transmission is discussed as another possible mechanism of avian bornavirus transmission since viral RNA was detected in eggs and embryos originating from infected birds of various species [8, 31–34]. However, proof of productive infection of the embryos by immunohistochemistry or virus isolation is still missing [8, 33]. This lack of convincing data is at least partly explained by the variable and usually very long incubation period of months or even years, requiring laborious infection experiments which will usually cover at least several months [5, 6, 8].

Only a decade ago, important steps towards unravelling the largely unknown epidemiology of the mammalian Borna disease virus 1 (BoDV-1) were made by applying phylogenetic analysis [35, 36]. The identification of five sequence clusters stably associated with their geographic origin within the endemic region in central Europe (S1 Fig) suggested the existence of rather immobile reservoir hosts. On the contrary, these observations argued against an important epidemiological role of known BoDV-1 hosts, such as horses, sheep and other agricultural animals, which are extensively traded. The epidemiological pattern was also considered incompatible with the existence of highly mobile avian reservoirs. Encouraged by these crucial results subsequent field studies focused on rodents and insectivores in the endemic region of BoDV-1. These efforts finally led to the detection of BoDV-1 in bicolored white-toothed shrews (*Crocidura leucodon*). In these animals the virus has a broad tissue distribution and infectious particles are shed with several excretions, making them a potential natural reservoir [4, 37–40].

In this study we used similar approaches in order to gain a better understanding of the epidemiology of avian bornavirus infections. To increase the number of bornavirus sequences available for phylogenetic analysis, samples were collected from wild or captive Passeriformes, Psittaciformes and aquatic birds in Germany and screened for the presence of bornavirus RNA by RT-PCR assays. Subsequently, phylogenetic analysis of newly identified bornavirus sequences was performed together with all related bornavirus sequences derived from GenBank.

## Material and Methods

### Samples collected from aquatic and passerine birds in Germany

Organ samples and cloacal swabs from aquatic birds and Passeriformes were provided by veterinarians, diagnostic laboratories and ornithologists in Germany. Aquatic bird samples were collected in 2009 to 2016 from a total of 738 wild (n = 706) or captive (n = 32) individuals belonging to six different orders (Anseriformes, Charadriiformes, Pelicaniformes, Gruiformes, Ciconiiformes and Sphenisciformes). The largest batch of samples was comprised of pooled lungs and intestines collected from 282 wild Eurasian oystercatchers (*Haematopus ostralegus*) found dead at the Wadden Sea coast of Schleswig Holstein during winter 2011/2012 [41]. Samples from 145 wild and captive Passeriformes, including 48 common canaries (*Serinus canaria* forma domestica), were collected during 2011 and 2016. Except for the Eurasian oystercatcher samples, the majority of tested organ samples were brains, but for few birds other organs, such as proventriculus or crop, were analyzed in addition to or instead of the brain. Analysis was performed either for individual samples or as pools of three to four samples. For pools which tested positive for bornaviruses, analysis was repeated individually for all samples included in the respective pool.

All animals were handled according to national and European legislation, namely the EU council directive 86/609/EEC for the protection of animals. Organ samples originated from perished animals and had been collected for diagnostic purposes. No animals were sacrificed as part of this study. Live free-ranging animals were captured and sampled as part of ring-banding projects. Licenses were obtained from the authorities of the respective federal states (Rhineland-Palatinate North: 425–105.1407; Rhineland-Palatinate South: 42/553-235). Handling and sampling of live animals was performed by trained personnel, with animal safety and comfort as the first priority during minimally invasive sampling using cloacal swabs.

### Detection of bornavirus RNA by conventional RT-PCR assays

Total RNA was extracted from swabs using QIAamp viral RNA mini kit (Qiagen, Hilden, Germany) and from homogenized organ samples by either RNeasy kit (Qiagen) or phenol-chloroform extraction with Trifast (Peqlab, Erlangen, Germany) as described previously [7, 8]. Reverse transcription (RT) was performed with 2 μg extracted RNA in a 20 μl reaction using Revertaid reverse transcription reagents (Thermo Scientific, St. Leon-Rot, Germany). Subsequently, samples were screened for bornavirus-derived RNA using three different RT-PCR assays in parallel. All samples were analysed with degenerate primer pairs Ncon and Mcon (S1 Table), which were demonstrated to detect a broad range of different mammalian and avian bornaviruses [8, 18, 26]. Aquatic bird samples were additionally tested with primer pair ABBV-1_M (S1 Table), which is essentially covering the same genomic region as the Mcon primer pair, but specifically targets the sequence of ABBV-1 [22]. Passerine samples were additionally tested with the degenerate primer pair Ccon (S1 Table), which was designed to detect bornaviruses of Passeriformes [8, 18]. Identities of PCR products were confirmed by Sanger sequencing (GATC, Cologne, Germany).

Furthermore, Sanger sequencing of overlapping PCR products was performed to determine partial bornavirus genome sequences from selected bornavirus-positive samples, including the coding-complete genome of ABBV-1 AF-168 from a Eurasian oystercatcher (GenBank accession number KU748788). Additional sequences were determined from 49 bornavirus-positive diagnostic samples originating from psittacines in Germany and the Netherlands during 2008 to 2015. Primers used for sequencing are listed in S1 Table. Bornavirus sequences generated during this study were submitted to GenBank with accession numbers KU748787 to KU748851.

### BLAST search for bornavirus sequences and phylogenetic analysis

BLAST search was performed to identify bornavirus sequences deposited in GenBank. For each of 14 avian bornaviruses a representative partial N and M gene sequence was used (corresponding to the products of the Ncon and Mcon primer pairs; positions 657 to 1003 and 1,932 to 2,241 of PaBV-2 genome FJ620690, respectively; Table 1). Hits with more than 90% nucleotide identity with the reference sequence were considered to belong to the same bornavirus. Duplicate sequences originating from the same individual were excluded from the analysis. Munia bornavirus 1 (MuBV-1) was not analysed, as at present only short fragments of raw sequence data of this virus are available in GenBank [8].

**Table 1:** Sequences of avian bornaviruses available in GenBank.

Species	Virus	reference sequence	number of sequences in GenBank	
			partial N gene ^a^	partial M gene ^b^
*Psittaciform 1 bornavirus*	PaBV-1	JX065207	4	4
	PaBV-2	FJ620690	52	35
	PaBV-3	FJ169440	2	2
	PaBV-4	JX065209	86	65
	PaBV-7	JX065210	1	1
*Psittaciform 2 bornavirus*	PaBV-5	KR612223	5	6
*Passeriform 1 bornavirus*	CnBV-1	KC464471	14	8
	CnBV-2	KC464478	10	7
	CnBV-3	KC595273	3	3
*Passeriform 2 bornavirus*	EsBV-1	KF680099	3	3
*Waterbird 1 bornavirus*	ABBV-1	KF578398	23	36 ^c^
	ABBV-2	KJ756399	1	1
unclassified	PaBV-6	FJ794726	-	2
	PaBV-8	KJ950621	7	4

^a^ product of Ncon PCR [12], corresponding to positions 657 to 1,003 (347 bp) of PaBV-2 #6609 (FJ620690) complete genome

^b^ product of Mcon PCR [12], corresponding to positions 1,932 to 2,241 (310 bp) of PaBV-2 #6609 (FJ620690) complete genome

^c^ corresponding to pos. 2,006 to 2,205 (200 bp) of PaBV-2 #6609 (FJ620690) complete genome

In total, BLAST search revealed 206 partial N gene and 177 partial M gene sequences, including the sequences generated during this study (Table 1). The most frequently sequenced avian bornaviruses PaBV-4, PaBV-2 and ABBV-1 were selected for individual phylogenetic analysis. Members of the viral species *Passeriform 1 bornavirus* (CnBV-1 to 3) were analysed together. For all other avian bornaviruses the available sequences were not sufficient to justify separate analysis. Additional phylogenetic trees were generated from complete P gene sequences of representative avian, mammalian and reptilian bornaviruses, 32 sequence fragments from members of the species *Psittaciform 1 bornavirus* covering partial N, complete X and P and partial M genes, as well as from 64 BoDV-1 sequences covering the complete N, X and P genes from naturally infected animals in the endemic regions in Germany, Switzerland and Liechtenstein.

Phylogenetic analysis was performed by Geneious R8 software (Biomatters Ltd., Auckland, New Zealand) using Neighbor-Joining algorithm and Jukes-Cantor distance model. Since not all sequences identified by BLAST search covered the Ncon or Mcon RT-PCR products completely, all sequences were trimmed to the length of the shortest sequence included in the respective analysis. All trees were rooted to selected *Bornaviridae* reference sequences. Maximum Likelihood (ML) analyses were used to confirm clustering of the nucleotide distance based trees. ML trees were calculated using a reduced alignment excluding the outgroup sequences, a General Time Reversible model and a complete deletion option in MEGA 6.0 (http://www.megasoftware.net/). Bootstrap support was assessed by 1,000 repetitive analyses.

## Results

### Clusters formed by ABBV-1 sequences from water birds in Europe are distinct from North American ABBV-1 sequences

Samples from a total of 738 waterbirds in Germany were tested for the presence of bornaviruses by three different RT-PCR assays. The analysis resulted in the detection of ABBV-1 RNA in four birds (Table 2), whereas no other avian or mammalian bornaviruses were detected. Cloacal swabs from three free-ranging mute swans (*Cygnus olor*) sampled in Munich in 2012 (KU748789) and in Hannover in 2013 (KU748790) and 2015 (KU748791) were positive. For the latter animal also the brain was available for analysis and likewise tested positive for the virus. In addition, ABBV-1 was detected in one out of 282 Eurasian oystercatchers (*H*. *ostralegus*), which were found dead during winter 2011/2012 at the Wadden Sea coast of Schleswig Holstein (bird AF-168; KU748788) [41]. Intestine, lung and whole blood from this bird were positive by RT-PCR, whereas the liver tested negative. This is in congruence with the detection of comparably low viral loads in the livers of bornavirus-infected psittacines [5, 42].

**Table 2:** Detection of ABBV-1 by RT-PCR from aquatic birds in Germany, 2009–2016.

Order	years of sampling	pos. / total samples	
Species		wild birds	captive birds
Charadriiformes			
Eurasian oystercatcher (*Haematopus ostralegus*)	2011–2012	1 / 282	-
common black-headed gull (*Larus ridibundus*)	2014–2016	0 / 63	-
others	2014–2015	0 / 8	-
Anseriformes			
mute swan (*Cygnus olor*)	2011–2016	3 / 71	-
Canada goose (*Branta canadensis*)	2014–2015	0 / 28	-
greylag goose / domestic goose (*Anser anser*)	2014–2015	0 / 61	0 / 2
Egyptian goose (*Alopochen aegyptiacus*)	2014–2015	0 / 87	-
mallard / domestic duck (*Anas platyrhynchos*)	2011–2015	0 / 71	0 / 2
others	2009–2016	0 / 31	0 / 19
other orders of aquatic birds ^a^	2013–2016	0 / 4	0 / 9
Total		4 / 706	0 / 32

^a^ Other aquatic bird orders tested were Pelicaniformes (n = 8), Gruiformes (n = 2), Ciconiiformes (n = 2) and Sphenisciformes (n = 1).

The specific primer pair ABBV-1_M proved to be most sensitive for ABBV-1 detection, giving clear bands for all positive samples. The degenerate primer pairs Mcon and Ncon failed to detect most of the positive samples, although both had been proven to reliably amplify cDNA from a broad range of avian and mammalian bornaviruses in previous studies [7, 8, 18].

**Fig 2:**
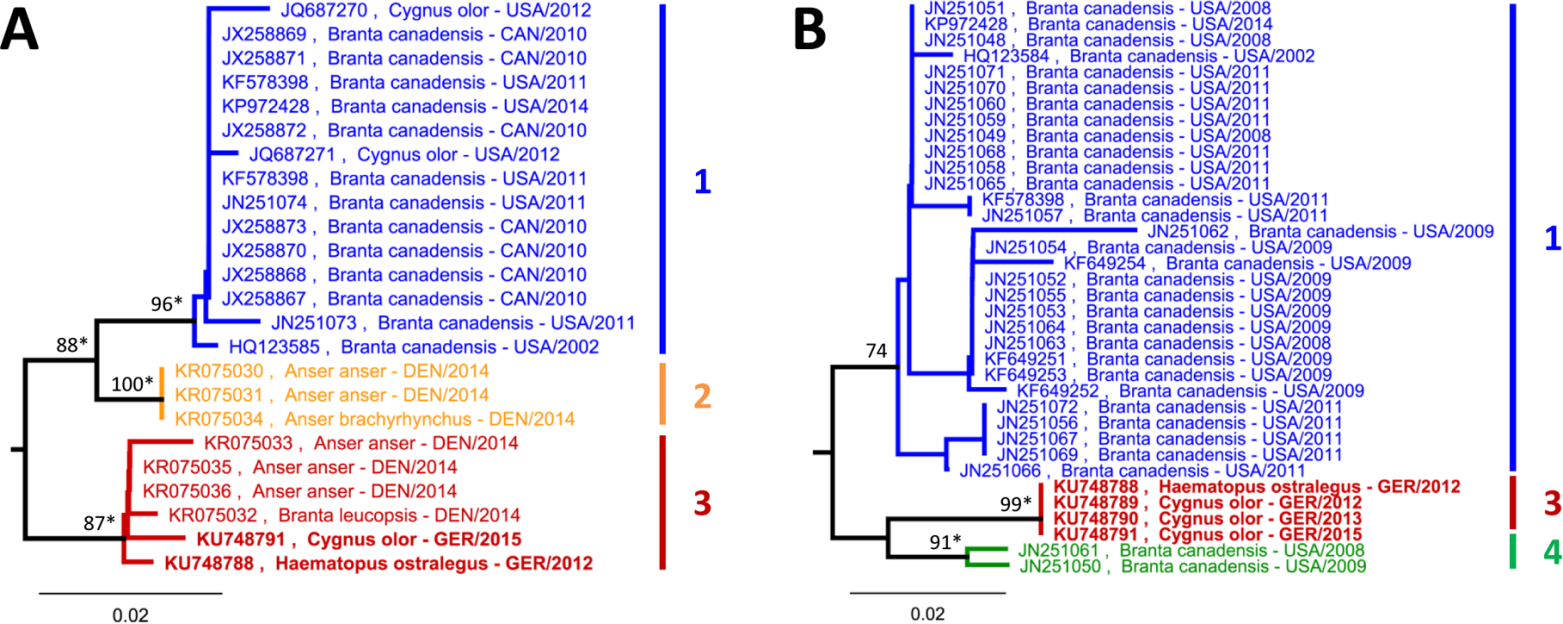
ABBV-1 sequences from wild aquatic birds form continent-associated clusters. Partial sequences of N (A, 320 bp) or M (B, 200 bp) genes of ABBV-1 were analysed. Phylogenetic analysis was performed using Neighbor-Joining algorithm and Jukes-Cantor distance model in Geneious R8. The trees were rooted with *Bornaviridae* references sequences (not shown; see Fig 1). Values at branches represent support in 1,000 bootstrap replicates. Only bootstrap values ≥70 at major branches are shown. Nodes with bootstrap support of ≥90 in additionally performed ML analysis are indicated by asterisks. Sequences depicted in bold were generated during this study. Clusters 3 and 4 were confirmed to be independent by alignment of short overlapping partial M gene sequences (113 bp) from both clusters (data not shown).

Partial M gene nucleotide sequences of the four positive samples were at least 99.4% identical among each other and at least 96.9% identical to ABBV-1 sequences from waterfowl in North America. The coding-complete genome sequence was determined for ABBV-1 AF-168 (KU748788) and shown to be 97.1 and 97.3% identical to two coding-complete ABBV-1 genomes from Canada geese (*Branta canadensis*) in the USA (KF578398, KP972428). Phylogenetic analysis of partial N and M gene sequences revealed four separate sequence clusters (Fig 2). Two of these clusters were composed of sequences originating from Canada geese and mute swans in the USA and Canada, whereas the two remaining clusters harboured sequences originating from Denmark and Germany. All four German ABBV-1 sequences were located in cluster 3 together with four sequences obtained from Danish geese (Fig 2).

It has to be noted, that the samples analysed in this study were not selected on a representative basis. Thus, our results do not allow prevalence calculation and comparison between species or sampling locations within Germany. This was beyond the scope of this study.

### PaBV-4 sequence clusters in captive psittacines are independent of geographical origin, time of sampling and host species

**Fig 3:**
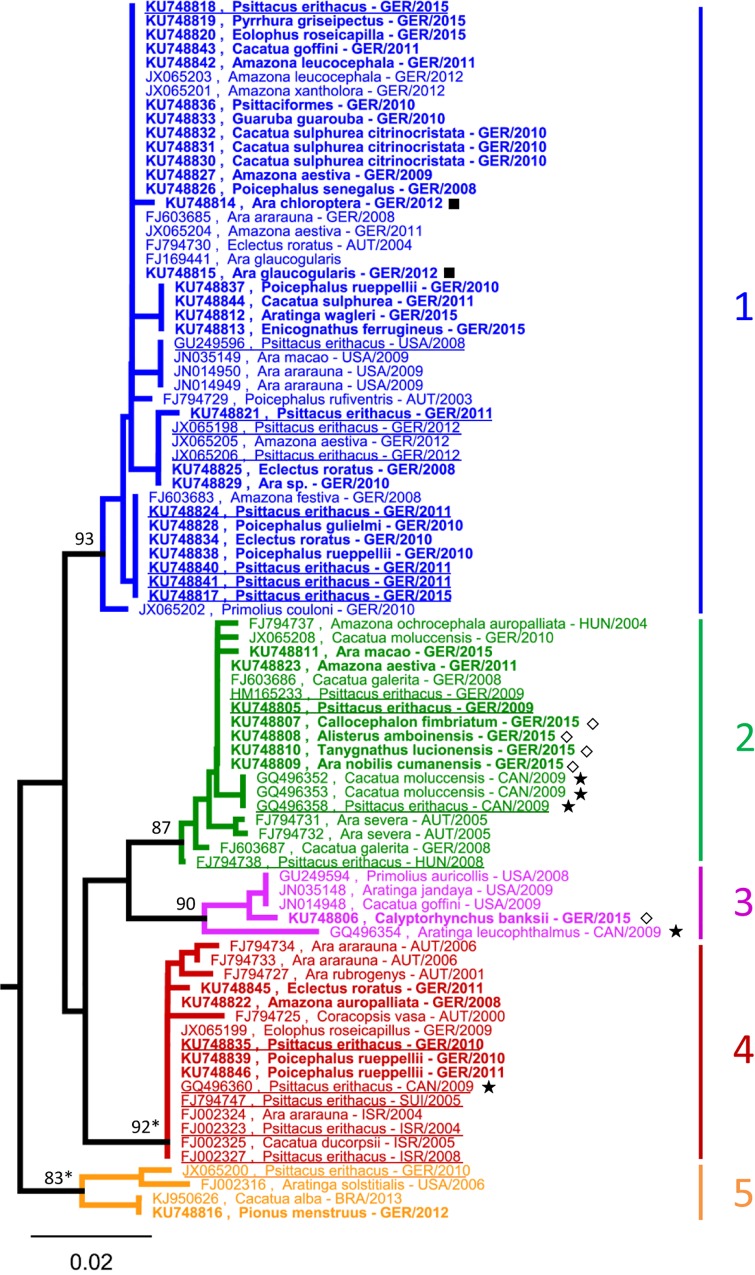
Clusters of PaBV-4 sequences from captive psittacines are not associated with regional origin or host species. Partial PaBV-4 N gene sequences (326 bp) were analysed using Neighbor-Joining algorithm and Jukes-Cantor distance model in Geneious R8. The tree was rooted with *Bornaviridae* references sequences (not shown; see Fig 1). Values at branches represent support in 1,000 bootstrap replicates. Only bootstrap values ≥70 at major branches are shown. Nodes with bootstrap support of ≥90 in additionally performed ML analysis are indicated by asterisks. Sequences depicted in bold were generated during this study. Sequences from African grey parrots (*Psittacus erithacus*) are underlined. (◇) flock A, Germany, 2015; (**★**) flock B, Canada, 2009 [43]; (■) flock C, Germany, 2012.

A total of 86 partial PaBV-4 N gene sequences of 326 bp length were available for analysis (Table 1). The minimal nucleotide identity among these sequences was 92.9%. Phylogenetic analysis identified at least five clearly separated clusters of PaBV-4 sequences, which were termed clusters 1 to 5 (Fig 3). Minimal nucleotide identity within each of these clusters was 97.1 to 98.8%.

More detailed analysis revealed no clear association of the clusters with host species, geographic origin and time of sampling (Fig 3). All five clusters contained European as well as North American sequences. In addition, four sequences from Israel were found in cluster 4 and a single Brazilian sequence in cluster 5. Interestingly, the Brazilian sequence (KJ950626) from an umbrella cockatoo (*Cacatua alba*) was closely related to a German sequence (KU748816) obtained from a blue-headed parrot (*Pionus menstruus*) which had been imported from Brazil shortly before sampling. Species association of the sequence clusters was analysed particularly for African grey parrots (*Psittacus erithacus*), since this was the most abundant host species in this set of sequences (n = 17). PaBV-4 sequences from African grey parrots were found in all clusters except for cluster 3 (Fig 3). Throughout all clusters, very closely related or even identical sequences were found in parallel in many different species, indicating that intra-species transmission is regularly accompanied by inter-species transmission in PaBV-4-infected captive psittacine populations.

**Fig 4:**
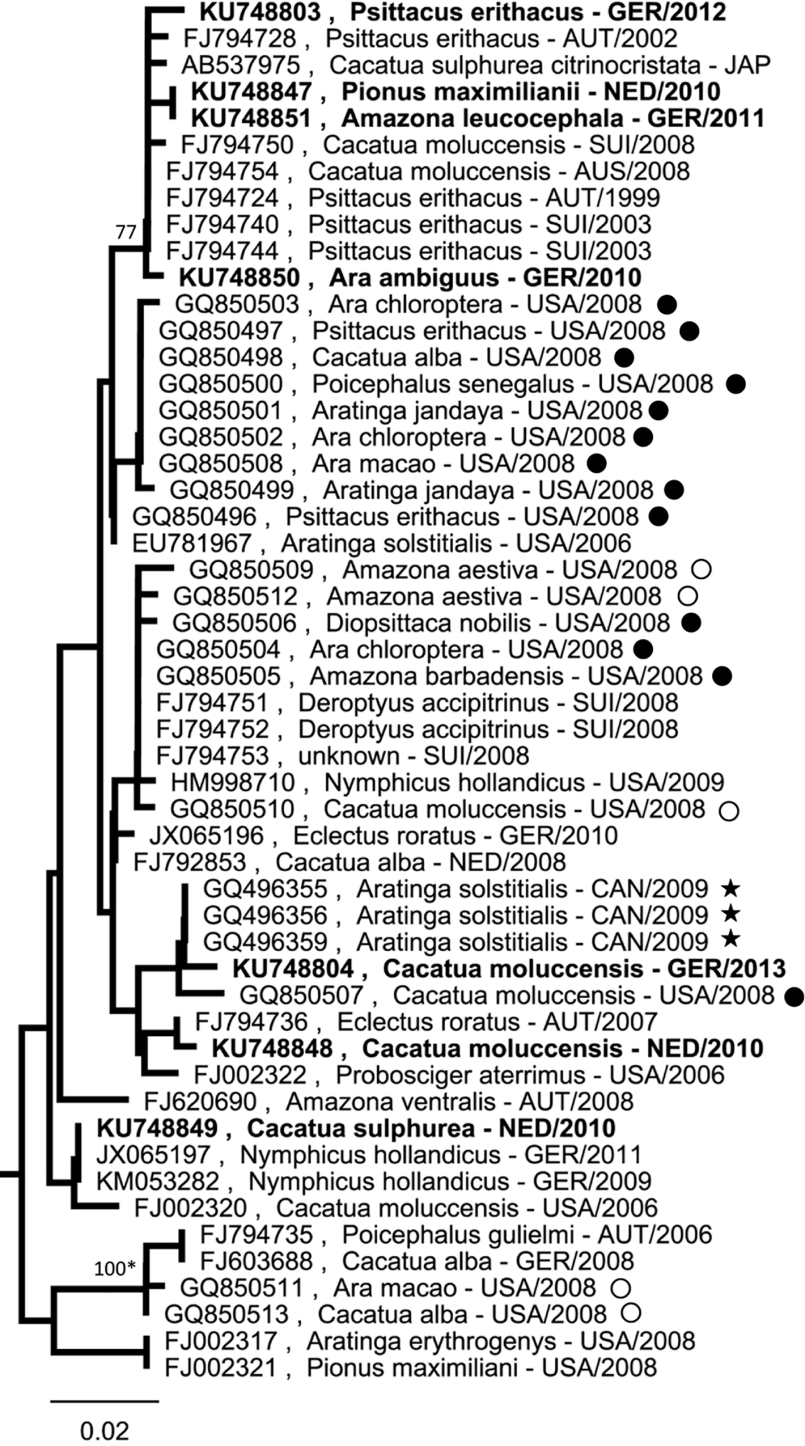
PaBV-2 sequences from captive psittacines do not form clear clusters. Partial PaBV-2 N gene sequences (342 bp) were analysed using Neighbor-Joining algorithm and Jukes-Cantor distance model in Geneious R8. The tree was rooted with *Bornaviridae* references sequences (not shown; see Fig 1). Values at branches represent support in 1,000 bootstrap replicates. Only bootstrap values ≥70 at major branches are shown. Nodes with bootstrap support of ≥90 in additionally performed ML analysis are indicated by asterisks. Sequences depicted in bold were generated during this study. (*) flock B, Canada, 2009 [43]; (●) flock D1, USA, 2008; (○) flock D2, USA, 2008; flocks D1 and D2 are epidemiologically linked [26].

Analysis of five PaBV-4 sequences originating from a single flock in Germany sampled in 2014 to 2015 (flock A) revealed four identical sequences (KU748807 to KU748810) belonging to cluster 2, while a fifth sequence (KU748806) possessed only 95.7% nucleotide identity with these sequences and was located in cluster 3 (Fig 3; S2 Fig). The comparably low sequence identity and the affiliation of these sequences to clearly distinct phylogenetic clades indicate that these viruses are very unlikely to have resulted from diversification within a single flock. To further exclude the remote possibility that cluster affiliation was an artefact due to the short sequence length, we performed an additional analysis with a set of 11 PaBV-4 sequences covering about one quarter of the viral genome (2,176 bp; S2 Fig). The analysis confirmed the separation of PaBV-4 into five clusters as well as the distinct cluster affiliation of the viruses detected in flock A. Thus, these findings strongly suggest that PaBV-4 was introduced into this flock on at least two occasions. Multiple introductions of bornaviruses were also observed for a psittacine colony previously described by Raghav and colleagues [43], in which at least three different PaBV-4 variants were present (flock B; Fig 3). In addition, also PaBV-2 was reported to be present in this bird collection (Fig 4). In contrast, two viruses detected in 2012 from another German flock (flock C) were genetically closely related (Fig 3; S2 Fig), so that origin from a common ancestor within the flock cannot be excluded. However, the fact that one of the birds (KU748815; *Ara glaucogularis*) was imported from Spain and sampled during quarantine whereas the other (KU748814; *Ara chloroptera*) had been in the flock already for three years likewise suggests the possibility of two independent introductions.

### PaBV-2 sequences from captive psittacines do not form clearly separated clusters

Phylogenetic analysis of PaBV-2 was performed using 52 partial N gene sequences (Table 1) with a minimal nucleotide identity of 94.2% among each other. In contrast to PaBV-4 these sequences did not divide into clearly separated clusters (Fig 4). Analysis of longer stretch of genomic sequences confirmed the remarkable lack of clear bottlenecks as compared to PaBV-4 (S2 Fig). However, closely related or identical PaBV-2 sequences found in different species confirmed observations from PaBV-4, further suggesting that horizontal inter-species transmission is frequent among bornavirus transmission events between Psittaciformes.

In a previous study, Kistler and colleagues [26] described the detection of 18 PaBV-2 sequences from two epidemiologically linked psittacine colonies with cases suggestive of PDD (flocks D1 and D2; Fig 4). At that time, the sequences were interpreted to be the result of a rapid spread of a single virus within and between the two flocks within few weeks. However, a retrospective analysis together with additional PaBV-2 sequences available today shows that the sequences from the two colonies are genetically rather diverse, suggesting either multiple virus introductions or long-lasting circulation of the virus during which the diversification occurred.

### Bornaviruses are frequently detected in common canaries but not in other tested Passeriformes

Samples from 31 wild and 114 captive Passeriformes were examined for the presence of bornaviruses. Sequences of CnBV-1, -2 or -3 were amplified from 11 out of 48 tested captive common canaries, but not from the remaining birds (Table 3).Other avian or mammalian bornaviruses were not detected in this sample collection.

**Table 3:** Bornavirus detection by RT-PCR from wild and captive Passeriformes in Germany, 2011–2016.

Family	year of sampling	pos. / total samples	
Species		wild birds	captive birds
True finches (Fringillidae)			
Common canary (*Serinus canaria* f. dom.)	2014–2016	-	11 / 48
others	2013–2016	0 / 14	0 / 32
Estrildid finches (Estrildidae)	2013–2016	-	0 / 28
Old World warblers (Sylviidae)	2011–2015	0 / 12	-
other Passeriformes families ^a^	2012–2015	0 / 5	0 / 6
Total		0 / 31	11 / 114

^a^ Other Passeriformes families tested were Thraupidae (n = 4), Muscicapidae (n = 3), Acrocephalidae, Cotingidae, Pycnonotidae, Regulidae (n = 1 each).

**Fig 5:**
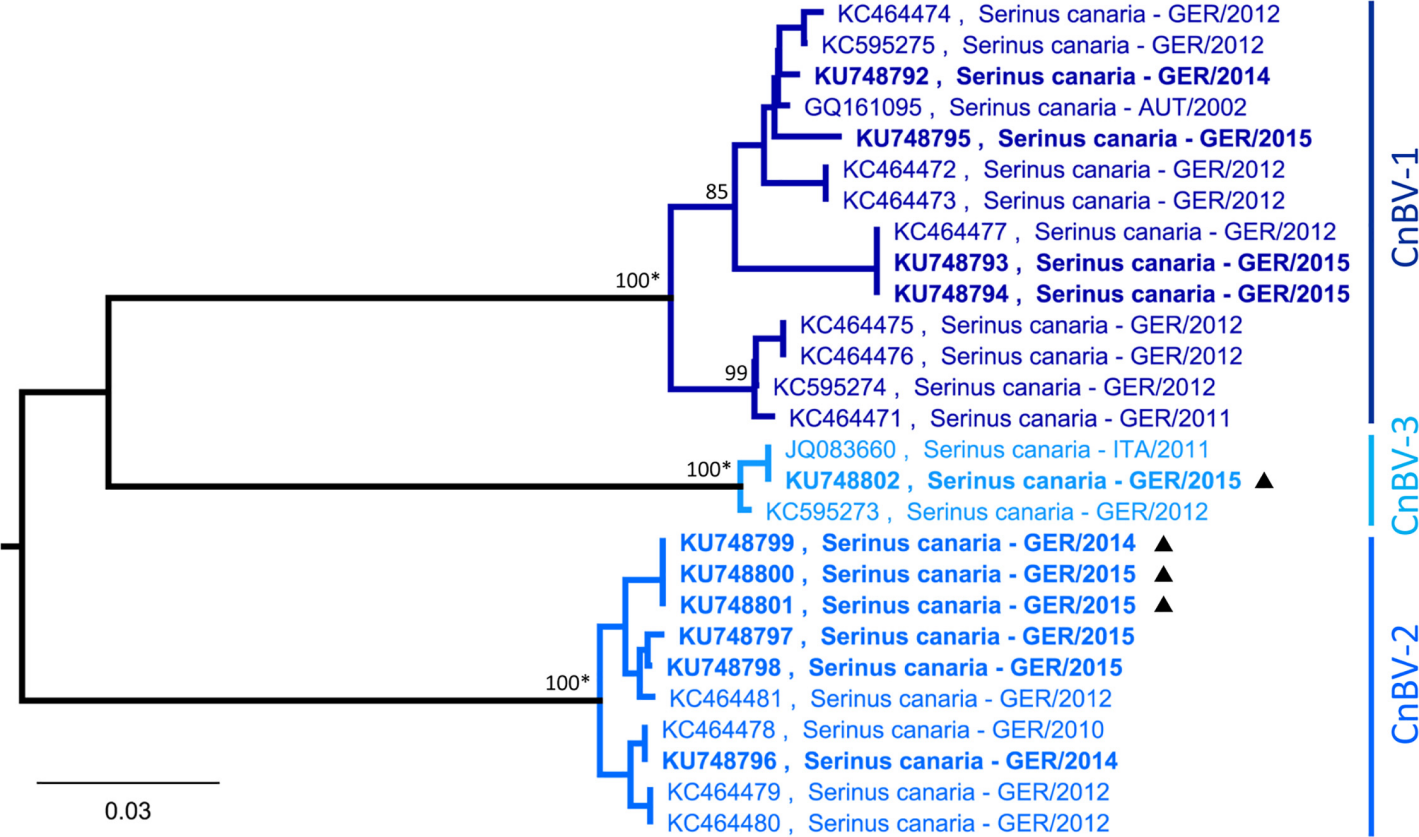
Clustering of CnBV-1, CnBV-2 and CnBV-3 detected in common canaries. Partial N gene sequences (343 bp) were analysed using Neighbor-Joining algorithm and Jukes-Cantor distance model in Geneious R8. The tree was rooted with *Bornaviridae* references sequences (not shown; see Fig 1). Values at branches represent support in 1,000 bootstrap replicates. Only bootstrap values ≥70 at major branches are shown. Nodes with bootstrap support of ≥90 in additionally performed ML analysis are indicated by asterisks. Sequences depicted in bold were generated during this study. (▲) flock E, Germany, 2014–2015.

In one mixed bird colony, four out of 16 canary samples were found to be positive for either CnBV-2 or CnBV-3 (flock E; Fig 5). Twelve samples of other passerines and three psittacine samples obtained from the same flock were negative for bornaviruses (data not shown). The wide bornavirus distribution in canaries as well as their apparently low prevalence in many other passerine species is in agreement with previous studies in which we detected canary bornaviruses in 26 out of 118 canaries [8], but only three out of 286 other passerine samples were positive for EsBV-1 [18]. However, it has to be noted that conclusions on bornavirus prevalence in individual species are not possible due to the low sample numbers for most species. Thus, additional passerine species with a high prevalence of bornavirus infections may have been missed in these studies.

Phylogenetic analysis was performed with 14, ten and three partial N gene sequences of CnBV-1, -2 and -3, respectively (Table 1; Fig 5). All available sequences originated from bornavirus-infected canaries in Europe, namely Germany (n = 25), Austria (n = 1) and Italy (n = 1). CnBV-1 sequences were relatively diverse with a minimal identity of 94.6%, while CnBV-2 sequences were more uniform showing at least 98.0% nucleotide identity among each other.

## Discussion

Since the discovery of the first avian bornaviruses from parrots suffering from PDD in 2008, at least 15 different bornaviruses have been detected in various avian host species [1, 2]. Despite the markedly extended knowledge on the biology of *Bornaviridae*, many aspects of epidemiology and phylogeny of these viruses remain unclear. This study was set out to summarize and extend the current knowledge on selected avian bornaviruses.

BLAST search using reference sequences of 14 avian bornaviruses yielded highest hit numbers for PaBV-4 and PaBV-2, which is consistent with the view that these viruses are most widely distributed in captive psittacine populations [7, 13, 14]. However, it is unknown to which extent the relative abundance of some bornaviruses and the rare occurrence of others among the sequences submitted to the databases is biased by the use of diagnostic tools. For instance, a widely used real-time PCR assay first described by Honkavuori and colleagues [11] was designed to specifically detect PaBV-4 and will likely miss other bornaviruses.

Phylogenetic analysis of the sequences from this study was performed together with those obtained from GenBank. The analysis revealed at least five and four clearly separated sequence clusters of PaBV-4 and ABBV-1, respectively, while no such clustering was observed for PaBV-2. PaBV-4 sequences from captive psittacines were not linked to geographic origin, time of sampling and host species. In contrast, a strong geographical association was demonstrated for ABBV-1 from wild aquatic birds. In the following sections the potential significance of these findings for the biology and epidemiology of avian bornaviruses will be discussed.

Avian bornaviruses are assumed to be transmitted via urofecal-oral route [7, 8, 28], but experimental data is equivocal and the exact mode of transmission remains unknown [5, 6, 8]. Overall, horizontal transmission appears to be rather inefficient, since birds exposed to naturally or experimentally infected cage mates remain virus-negative for months or even years [5, 30]. Likewise, vertical transmission is discussed as a potentially important transmission route of avian bornaviruses, but final proof for this hypothesis is still missing [8, 31–34]. A dominant role of vertical transmission would be expected to result in a strong association of sequences with host species. In this study, PaBV-4 sequence clusters were found not to be associated with particular psittacine species and identical sequences were identified to originate from various different hosts. Similarly, identical PaBV-2 sequences were obtained in many cases from divergent psittacine species. These findings suggest that horizontal inter-species transmission is relatively frequent among overall PaBV-2 and PaBV-4 transmission events. Thus, our observations emphasize the importance of horizontal transmission as a driving force of bornavirus spread in captive psittacine populations. However, the results do not exclude the possibility of horizontal and vertical transmission modes existing in parallel. Investigating the mechanisms of horizontal and vertical transmission of avian bornaviruses will have to be important issues of future bornavirus research.

In addition to gaining information on bornavirus transmission in general, phylogenetic analysis may be used also for the investigation of bornavirus spread within or between particular flocks. Here we identified a flock of mixed psittacine species into which PaBV-4 strains of different clusters had been introduced on at least two independent occasions. In addition, our analysis retrospectively indicated multiple introductions of either PaBV-2 or PaBV-4 into two previously described psittacine populations [26, 43].

Psittacine bornaviruses, such as PaBV-2 and PaBV-4, were so far almost exclusively found in captive birds, while only little is known about their prevalence in free-ranging Psittaciformes or other wild birds. The apparent lack of geographic association of bornavirus sequences from captive psittacines presumably reflects viral distribution through extensive trading of these birds in the past, leading to a nearly global distribution of various genetic variants. In contrast, the five BoDV-1 clusters display a remarkable geographical association, which is in agreement with the restricted mobility of wild bicolored white-toothed shrews, the assumed natural reservoir of this bornavirus [35–40]. Similarly, ABBV-1 in wild waterfowl forms geographically separated clusters originating from either Europe or North America. However, in congruence with the high mobility of these hosts, ABBV-1 clusters possess much more extended dispersal areas as compared to BoDV-1 clusters. The clear clustering suggests viral spread between the two continents to be a rare event.

The wild reservoirs of parrot bornaviruses and their introduction into captive psittacine populations are not yet understood. As a consequence, also the origins of the PaBV-4 clusters as well as possible explanations for the apparent lack of clear bottlenecks during PaBV-2 diversification remain elusive. Similar to ABBV-1 and BoDV-1, PaBV-4 may have split up into several possibly geographically segregated clusters already in its unknown wild reservoir. Alternatively, each cluster currently found may represent the descendants of one or few independent introductions into the captive psittacine population. Further information about the prevalence of these viruses in wild birds is required to answer these questions. Encinas-Nagel and colleagues [44] reported the detection of low levels of PaBV-4-specific RNA or bornavirus-reactive antibodies in wild psittacine birds submitted to rehabilitation centres in Brazil. However, most of these birds had been in these centres for several weeks or months before sampling, questioning that these results are representative for free-ranging populations. Sequences enabling a more detailed analysis of the findings were not provided [44]. More recently, a Japanese study reported the detection of PaBV-2 and PaBV-4 from wild birds of various non-psittaciform orders, including Passeriformes and Anseriformes [45]. These findings are in remarkable contrast to the results presented here and in previously published studies on Passeriformes and Anseriformes in Europe and North America [8, 18, 20–22]. In these studies psittacine bornaviruses were not detected in wild or captive Passeriformes or Anseriformes although the employed PCR assays, such as Ncon, Mcon or ABBV-1_M, are generally able to amplify these viruses [8, 18]. Moreover, all wild bird sequences from Japan are identical or closely related to sequences obtained from captive psittacines tested in the same laboratory. The authors considered discrete sequence variations to rule out the possibility of contamination [45]. However, a more detailed analysis revealed these PaBV-2 and PaBV-4 sequences to contain untypically high proportions of non-synonymous mutations (data not shown), suggesting that they may be laboratory artefacts rather than naturally occurring sequence variants.

The possibility of natural infections of wild birds with mammalian BoDV-1 is another subject of controversies. Berg and colleagues [46] suggested Anseriformes and Passeriformes to be natural reservoirs of BoDV-1 based on positive PCR results obtained from faecal samples of a mallard (*Anas plathyrynchos*) and a jack daw (*Corvus monedula*) from Sweden. Retrospective phylogenetic analysis placed both sequences in BoDV-1 cluster 4 which is normally found in Central and Southern Germany (Lower Saxony to Bavaria) [35]. To date, the role of wild birds as a reservoir of BoDV-1 still lacks independent confirmation. In our own studies we did not detect BoDV-1 or BoDV-2 in avian samples despite the use of the Ncon and Mcon RT-PCR assays, which are able to detect both viruses [7–9, 18].

Bornaviruses of psittacines and aquatic birds were demonstrated to spread between avian species or even orders and for many of these viruses a world-wide distribution was found. In contrast, all CnBV-1, 2 and 3 isolates known to date originate exclusively from common canaries in Europe. Their geographic restriction to Europe and mainly Germany results most likely from the fact that corresponding studies from other parts of the world have not been reported to date. In contrast, the exclusive detection of these viruses in common canaries but not in other passerine species is less likely an effect solely of sampling biases. In this and a previous study we were not able to detect canary bornaviruses in a total of 383 captive and free-ranging passerines other than common canaries despite the use of suitable PCR assays [18]. The viruses were not even detected in birds housed together with infected canaries. Although we cannot exclude that other frequent hosts of these viruses may have been missed due to underrepresentation in the sample collections, these observations indicate that the canary bornaviruses may have a more restricted natural host range than other avian bornaviruses. Whether a more dominant role of vertical transmission compared to horizontal transmission contributes to the supposedly stronger host specificity of these viruses remains to be elucidated.

In summary we gained important insights into the epidemiology and biology of avian bornaviruses. Our results provide a basis for further research, such as investigating bornavirus transmission routes or searching for their natural reservoirs. Furthermore, we provided information useful to improve interpretation of sequence data obtained from prevalence studies or case reports. We would like to encourage researchers to routinely compile and submit high quality sequence data obtained from bornavirus-infected individuals to support a better understanding of this interesting and clinically important virus family.

## Supporting Information

S1 Fig BoDV-1 forms geographically associated clusters in endemic regions in central Europe. Complete BoDV-1 N, X and P gene sequences from naturally infected shrews and agricultural animals in Germany, Switzerland and Liechtenstein were analysed together with sequences of widely used laboratory strains. Phylogenetic trees were build using Neighbor-Joining algorithm and Jukes-Cantor distance model in Geneious R8 and rooted with sequence BoDV-2 No/98 (AJ311524; not shown). Values at branches represent support in 1,000 bootstrap replicates. Only bootstrap values ≥70 at major branches are shown. Nodes with bootstrap support of ≥90 in additionally performed ML analysis are indicated by asterisks. Germany (GER): BA = Bavaria, BW = Baden-Wuerttemberg, HE = Hesse, LS = Lower Saxony, SA = Saxony-Anhalt; Switzerland (SUI): GR = Grisons, SG = St. Gall; Liechtenstein = L. Cluster designations and information on hosts and geographic origin were adapted from Durrwald et al. [39].
(TIF)

S2 Fig Comparative analysis of *Psittaciform 1 bornavirus* sequences indicates marked bottlenecks within the evolution of PaBV-4 but not PaBV-2. Partial genome sequences (2,176 bp) of members of the species *Psittaciform 1 bornavirus* (PaBV-1. 2. 3. 4, and 7) were analysed using Neighbor-Joining algorithm and Jukes-Cantor distance model in Geneious R8. The tree was rooted with *Bornaviridae* references sequences (not shown; see Fig 1). Values at branches represent support in 1,000 bootstrap replicates. Only bootstrap values ≥70 at major branches are shown. Sequences depicted in bold were generated during this study. (◇) flock A, Germany, 2015; (■) flock C, Germany, 2012.
(TIF)

S1 Table Primers used for detection and sequencing of bornaviruses in this study. (PDF)
