# Supplementary figures and images for: Phylogenetic Analysis Supports Horizontal Transmission as a Driving Force of the Spread of Avian Bornaviruses
Source: PLoS One. 2016 Aug 18;11(8):e0160936. doi: 10.1371/journal.pone.0160936 (PMC4990238; doi:10.1371/journal.pone.0160936)

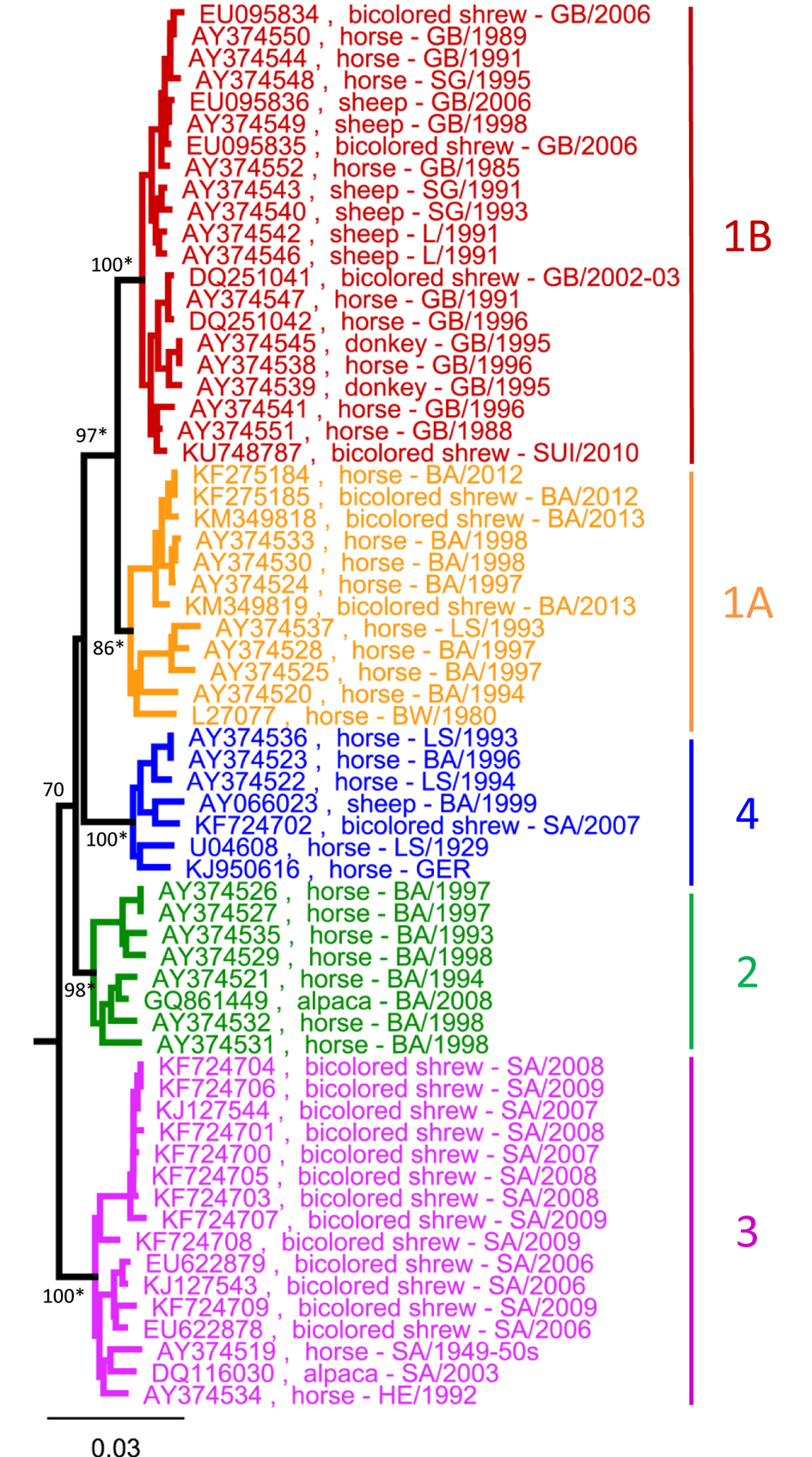

Supplement: S1 Fig — Complete BoDV-1 N, X and P gene sequences from naturally infected shrews and agricultural animals in Germany, Switzerland and Liechtenstein were analysed together with sequences of widely used laboratory strains. Phylogenetic trees were build using Neighbor-Joining algorithm and Jukes-Cantor distance model in Geneious R8 and rooted with sequence BoDV-2 No/98 (AJ311524; not shown). Values at branches represent support in 1,000 bootstrap replicates. Only bootstrap values ≥70 at major branches are shown. Nodes with bootstrap support of ≥90 in additionally performed ML analysis are indicated by asterisks. Germany (GER): BA = Bavaria, BW = Baden-Wuerttemberg, HE = Hesse, LS = Lower Saxony, SA = Saxony-Anhalt; Switzerland (SUI): GR = Grisons, SG = St. Gall; Liechtenstein = L. Cluster designations and information on hosts and geographic origin were adapted from Durrwald et al. [39]. (TIF) [file pone.0160936.s001.tif]

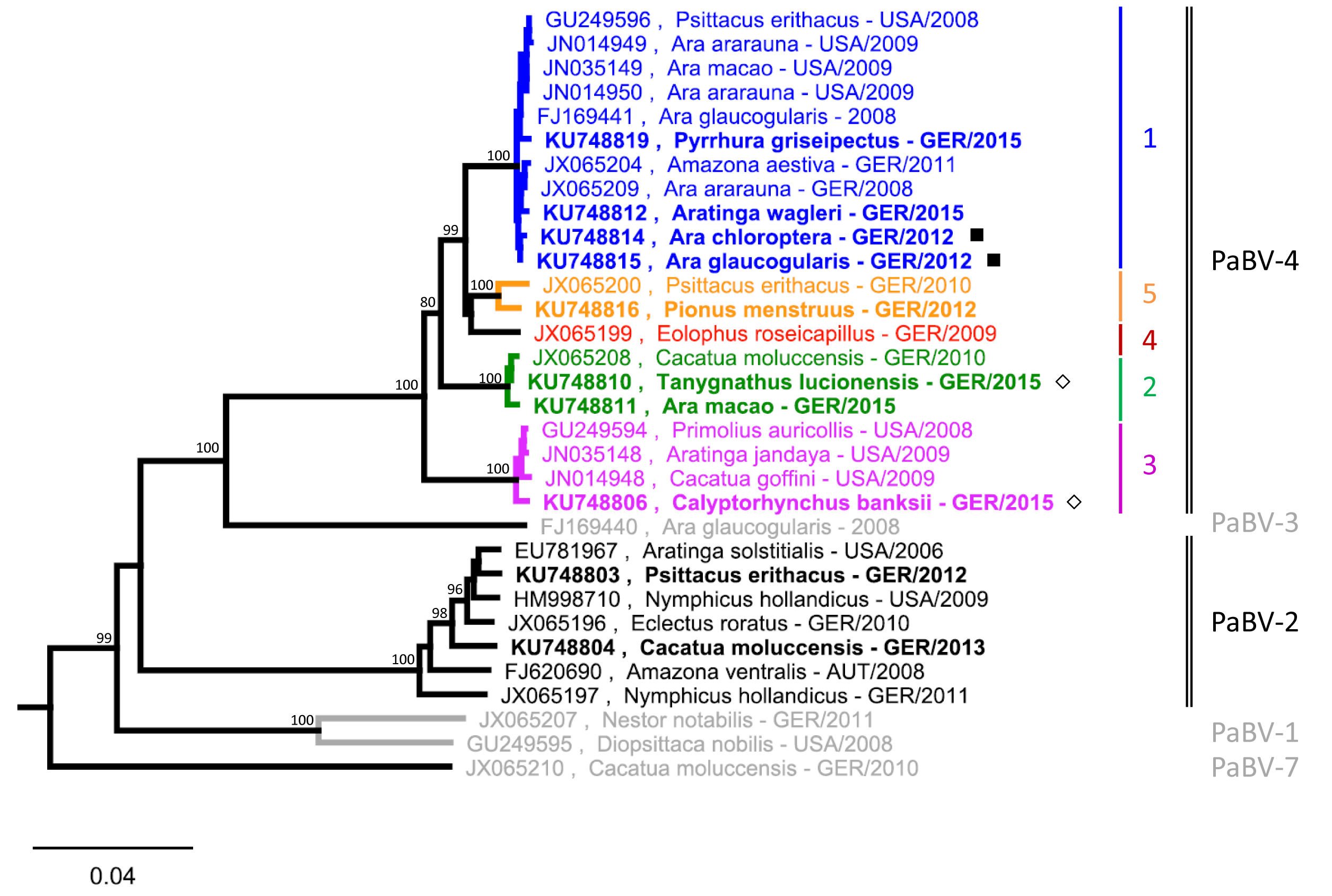

Supplement: S2 Fig — Partial genome sequences (2,176 bp) of members of the species Psittaciform 1 bornavirus (PaBV-1. 2. 3. 4, and 7) were analysed using Neighbor-Joining algorithm and Jukes-Cantor distance model in Geneious R8. The tree was rooted with Bornaviridae references sequences (not shown; see Fig 1). Values at branches represent support in 1,000 bootstrap replicates. Only bootstrap values ≥70 at major branches are shown. Sequences depicted in bold were generated during this study. (◇) flock A, Germany, 2015; (■) flock C, Germany, 2012. (TIF) [file pone.0160936.s002.tif]
