# Supplementary material for: Phylogenetic Analysis Supports Horizontal Transmission as a Driving Force of the Spread of Avian Bornaviruses
Source: PLoS One. 2016 Aug 18;11(8):e0160936. doi: 10.1371/journal.pone.0160936 (PMC4990238; doi:10.1371/journal.pone.0160936)
Supplement: S1 Table — (PDF) [file pone.0160936.s003.pdf]

S1 Table. Primers used for detection and sequencing of bornaviruses in this study

Assay	Primer	Sequence (5' to 3')	Reference
Ncon	Ncon-F	CCHCATGAGGCTATWGATTGGATTAACG	[1]
	Ncon-R	GCMCGGTAGCCNGCCATTGTDGG	[1]
Mcon	Mcon-F	GGRCAAGGTAATYGTYCCTGGATGGCC	[1]
	Mcon-R	CCAACACCAATGTTCCGAAGMCG	[1]
ABBV-1_M	ABBV-1_1990+ [a]	GGTAATTGTTCCTGGATGGC	[2]
	ABBV-1_2322- [a]	ACACCAATGTTCCGAAGACG	[2]
Ccon	Ccon-F	GGTGTGGTGATTGGKTCTTC	[3]
	Ccon-R	SGGYGAYTCAAAGTCTGTAG	[3]
Sequencing	ABBV-1_634+	CCTCATGAGGCTATTGATTGG	[2]
	ABBV-1_991-	AGTAGAATGCCGCAGAAGC	[2]
	ABBV-1_2876+	TAGGCGGTCATATCCTCTTGTG	this study
	ABBV-1_3108-	GCAATCTTCCCATGCATCTC	this study
	ABBV-1_3934+	TGGTGTCCTCATTAGATCCGC	this study
	ABBV-1_4134-	GGTCCAATATCAGCCTCCCG	this study
	ABBV-1_4905+	AGGCTGTTATTAATTCAAGACGTG	this study
	ABBV-1_5091-	ACGGAGCCCTTGTAAAACGG	this study
	ABBV-1_5870+	AGAGTCACTGATTCAACAGGTG	this study
	ABBV-1_6047-	CTTGCATTATTGAGGGCGGC	this study
	ABBV-1_6931+	AACCAGAGCTGCTAACCTTG	this study
	ABBV-1_7083-	CCACCTTGTATATTTGGACGGAC	this study
	ABBV-1_8019+	ATTCGCTGGCAGGTAAAGGG	this study
	ABBV-1_8190-	GTAGGGTCGCCTGCTAACAC	this study
	CnBV-1_834-	CTGCGGGAATTGTTAGTGTC	this study
	CnBV-1_1455+	CCAAAGGACAGGAAGGGAGC	this study
	CnBV-1_1466-	CCTGTCCTTTGGTCTGGGTC	this study
	CnBV-1_2291-	GGACCAATGCCAATATTCCGC	this study
	CnBV-2_592-	AGCTGGCCTATTGAGTGATG	this study
	CnBV-2_1314-	GTACTGTCTGCGGGTCTTCC	this study
	CnBV-2_1475+	CAAGCAACTCGTGACTGAGC	this study
	CnBV-2_2310-	GCGAATGTCAGGTGCAAGAG	this study
	CnBV-3_725-	TCCATGAATTCCTTCCCGGG	this study
	CnBV-3_1526-	TTTTCCGCCAGCTCAGTGAC	this study
	CnBV-3_2321-	GAGCCTGAGGTGCGGATATC	this study
	PaBV-2_1+ [b]	TGTTGCGGTAACAACCAAC	this study
	PaBV-2_500-	CCCCAATAAGGAGTGAACAGC	this study
	PaBV-2_1183-	GTTCATTAGTTTGCAAATCC	this study
	PaBV-2_1765+	CTGCAACGATGCTTCCCTC	this study
	PaBV-2_2293-	TGGAATGGCGTATGTCTGGG	this study
	PaBV-2_8914- [b]	GCGCTACAACAAAACCAC	this study
	PaBV-4_838-	GCTACAGCCGGAATTGTTAG	this study
	PaBV-4_1236-	CCAGCAATGTCAATTCCAAG	this study
	PaBV-4_1569+	AGGAGCCAAACTGGAAACAG	this study

[a] Primers can be used also for sequencing of PaBV-4.

[b] Due to the highly conserved genome ends, these primers can be used for all known members of the genus *Bornavirus*.

References:

1. Kistler AL, Gancz A, Clubb S, Skewes-Cox P, Fischer K, Sorber K, et al. Recovery of divergent avian bornaviruses from cases of proventricular dilatation disease: identification of a candidate etiologic agent. Virol J. 2008;5:88. PubMed PMID: 18671869.

2. Payne S, Covaleda L, Jianhua G, Swafford S, Baroch J, Ferro PJ, et al. Detection and characterization of a distinct bornavirus lineage from healthy Canada geese (*Branta canadensis*). J Virol. 2011;85(22):12053-6. PubMed PMID: 21900161.

3. Rubbenstroth D, Rinder M, Stein M, Höper D, Kaspers B, Brosinski K, et al. Avian bornaviruses are widely distributed in canary birds (*Serinus canaria* f. domestica). Vet Microbiol. 2013;165(3-4):287-95. Epub 2013/05/02. doi: 10.1016/j.vetmic.2013.03.024. PubMed PMID: 23631925.
